# A Novel Liposome-Based Adjuvant CAF01 for Induction of CD8^+^ Cytotoxic T-Lymphocytes (CTL) to HIV-1 Minimal CTL Peptides in HLA-A*0201 Transgenic Mice

**DOI:** 10.1371/journal.pone.0006950

**Published:** 2009-09-11

**Authors:** Gregers Jacob Gram, Ingrid Karlsson, Else Marie Agger, Peter Andersen, Anders Fomsgaard

**Affiliations:** 1 Department of Virology, Statens Serum Institut, Copenhagen, Denmark; 2 Department of Infectious Disease Immunology, Statens Serum Institut, Copenhagen, Denmark; New York University School of Medicine, United States of America

## Abstract

**Background:**

Specific cellular cytotoxic immune responses (CTL) are important in combating viral diseases and a highly desirable feature in the development of targeted HIV vaccines. Adjuvants are key components in vaccines and may assist the HIV immunogens in inducing the desired CTL responses. In search for appropriate adjuvants for CD8^+^ T cells it is important to measure the necessary immunological features e.g. functional cell killing/lysis in addition to immunological markers that can be monitored by simple immunological laboratory methods.

**Methodology/Principal Findings:**

We tested the ability of a novel two component adjuvant, CAF01, consisting of the immune stimulating synthetic glycolipid TDB (Trehalose-Dibehenate) incorporated into cationic DDA (Dimethyldioctadecylammonium bromide) liposomes to induce CD8^+^ T-cell restricted cellular immune responses towards subdominant minimal HLA-A0201-restricted CTL epitopes from HIV-1 proteins in HLA-A*0201 transgenic HHD mice. CAF01 has an acceptable safety profile and is used in preclinical development of vaccines against HIV-1, malaria and tuberculosis.

**Conclusions/Significance:**

We found that CAF01 induced cellular immune responses against HIV-1 minimal CTL epitopes in HLA-A*0201 transgenic mice to levels comparable with that of incomplete Freund's adjuvant.

## Introduction

Generation of cellular immune responses are of high significance in development of HIV-1 vaccines since the breath and expansion of cytotoxic T-lymphocytes (CTL) is associated with virus control [1], [2], [3]. To meet the high HIV-1 variation it is preferred for a vaccine to include several antigens or epitopes. After selection of vaccine epitope peptides, it is important to find an appropriate adjuvant capable of inducing a strong cellular immune response. In this search it is important to look for the necessary immunological features e.g. cell killing/lysis and not just immunological markers that can be monitored by simple immunological laboratory methods. Not many adjuvants are accessible for human use, and those, which can be used in laboratory animal experiments, are often incompatible with clinical trials in humans.

CAF01 is a novel adjuvant which has an acceptable safety profile and proved successful in preclinical development of antibody and Th cell activating vaccines against malaria and tuberculosis and are currently in clinical trials [4], [5]. Moreover, CAF01 induces very robust memory T cell responses that are maintained at high levels for >1 year postimmunization using a tuberculosis subunit vaccine [6]. CAF01 consists of DDA and TDB. DDA is a synthetic amphiphilic lipid compound comprising a hydrophilic positively charged dimethylammonium head–group attached to two hydrophobic 18-carbon alkyl chains (tail). In an aqueous environment, DDA self-assemble into closed vesicular bilayers (liposomes). The adjuvant efficacy and stability of the liposomes (DDA) is increased by incorporation of the synthetic glycolipid TDB (trehalose 6,6′-dibehenate) which is a synthetic analogue to the immune stimulatory component of the mycobacterial cell wall often referred to as the cord factor or trehalosedimycolate [5], [7], [8].

To induce additional CTL immunity during chronic HIV-1 infection by a therapeutic vaccination we have identified infrequently targeted but conserved HLA-A0201-restricted epitopes from Gag, Pol, Env, Vpu and Vif [9], [10], [11]. These relatively immune silent subdominant epitopes were modified as anchor-optimized peptides to improve immunogenicity for a vaccine. During development of our CTL inducing therapeutic HIV-1 vaccine in HLA-A*0201 transgenic mice, we have used the powerful standard incomplete Freund's adjuvant (IFA) which has, however, limited usability in humans [9], [10].

Here we show that CAF01 induced also functional CD8^+^ T cell immune responses against HIV-1 minimal CTL epitopes in HLA-A*0201 transgenic mice to levels comparable with or better than that of IFA.

## Results

### Novel adjuvant CAF01 helps inducing CTL similar to incomplete Freund's adjuvant

We compared the ability of the novel adjuvant CAF01 to induce T-cell responses in HLA-A*0201 transgenic HHD mice [12] using IFN-γ ELISPOT and ^51^Cr-release cytolytic assay after subcutaneous (s.c.) immunization with a subdominant 9-mer epitope Vif_101_ (GLADQLIHL) [10] together with the CD4^+^ T-helper epitope, PADRE [13]. The novel adjuvant CAF01 was compared with incomplete Freund's adjuvants (IFA).

**Figure 1:**
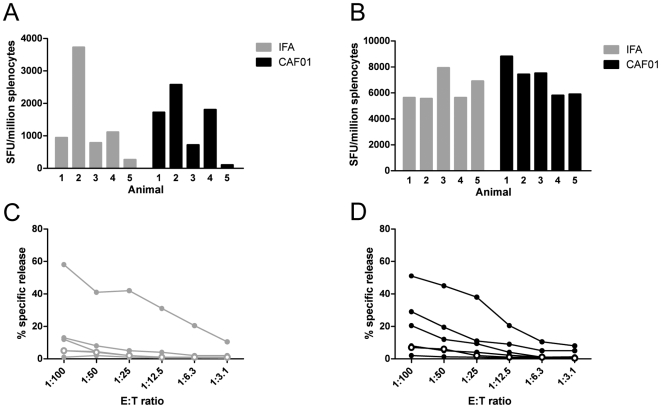
Cellular immune responses are induced to a similar level after immunization with IFA and CAF01. IFN-γ ELISPOT responses against (A) HLA-A2-restricted CD8 T cell epitope Vif_101_ and (B) PADRE Th epitope of splenocytes from HLA-A*0201 transgenic HHD mice after 10 days of s.c. immunization with Vif_101_ and PADRE in either IFA (grey bars, n = 5) or CAF01 adjuvants (black bars, n = 5) and 5 days *in vitro* prestimulation with individual epitopes. The background of an unimmunized mouse is substracted. Specific ^51^Cr-release from target cells preloaded with Vif_101_ after incubation with effector splenocytes from Vif_101_ and PADRE immunized mice adjuvanted with (C) IFA (grey, n = 5) or (D) CAF01 (black, n = 5). The percentage of specific lysis was calculated as 100×(experimental release-spontaneous release)/(total release-spontaneous release). Background lysis from an unimmunized mouse is shown (open circles). Significant positive levels are considered for >10% lysis at a 50∶1 ratio of effector:target (E:T) cells. One representative experiment out of three. SFU, spot forming units.

We found that both the HLA-A2-restricted CD8 T-cell epitope Vif_101_ and the CD4 Th epitope PADRE induced high responses in IFN-γ ELISPOT in both the IFA and CAF01 groups (Figure 1A+B). After 5 days of *in vitro* peptide prestimulation of splenocytes significant responses to Vif_101_ stimulation and PADRE stimulation (Figure 1A+B). The Vif_101_ or the PADRE responses were not significantly different between the IFA and CAF01 groups (t-test, p>0.05).

Splenocytes stimulated 5 days *in vitro* with Vif_101_ or the PADRE peptide were analyzed for the ability to lyse peptide-loaded HHD HLA-A*0201 target cells in a ^51^Cr-release cytolytic assay (Figure 1C+D). We found that in the groups of animals adjuvanted with IFA only one mouse significantly lysed Vif_101_-loaded target cells (Figure 1C), whereas three out of five mice adjuvanted with CAF01 significantly lysed Vif_101_ loaded target cells (Figure 1D). The CTL responses correlated with the ELISPOT responses in the sense that the highest responses in the IFN-γ ELISPOT were those which gave also responses in the ^51^Cr-release cytolytic assay (data not shown). Thus, CAF01 adjuvants supportetd CD8 T cell responses as good as or better than IFA.

### Including CD4^+^ T-helper epitope increases CTL responses

In order to evaluate the effect of including a CD4^+^ T-helper epitope (PADRE) with the CAF01 adjuvants for the induction of a CD8^+^ T cell response towards a minimal subdominant CD8^+^ T cell epitope (Vif_101_) we immunized HLA-A*0201 transgenic HHD mice subcutaneously (s.c.) with Vif_101_ with or without PADRE. The novel adjuvant CAF01 was used in both immunizations. IFN-γ ELISPOT and a ^51^Cr-release cytolytic assay was used to evaluate the T cell responses after five days *in vitro* stimulation.

**Figure 2:**
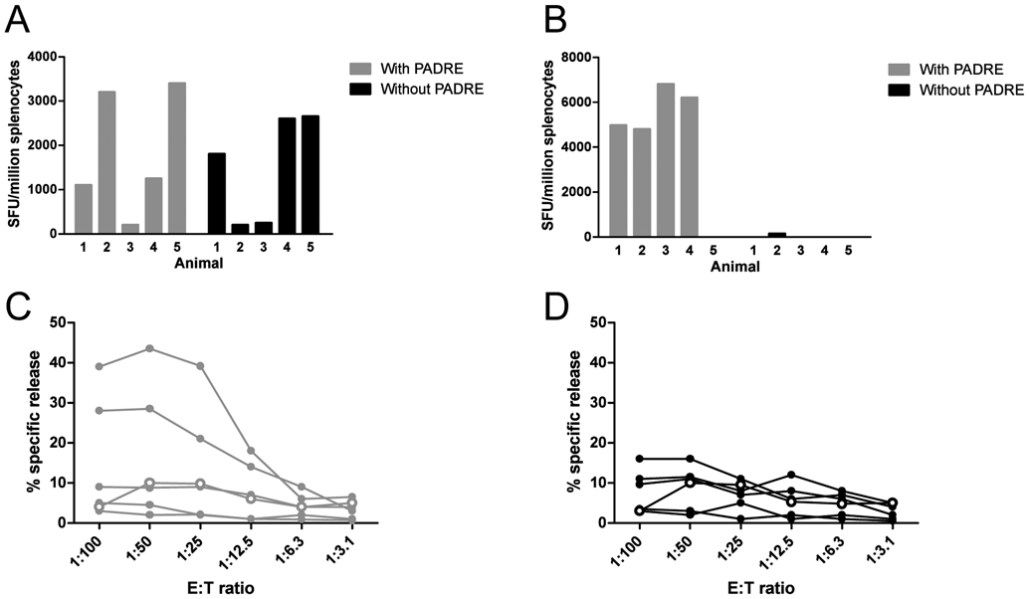
Cellular immune responses induced after immunization in CAF01 with or without a T helper epitope. IFN-γ ELISPOT responses against (A) Vif_101_ CTL epitope and (B) PADRE Th epitope of splenocytes from HLA-A*0201 transgenic HHD mice after 10 days of s.c. immunization with Vif_101_ and PADRE (grey bars, n = 5) or with Vif_101_ alone (black bars, n = 5) and 5 days *in vitro* prestimulation with individual epitopes. The background of an unimmunized mouse is substracted. Specific ^51^Cr-release from target cells preloaded with Vif_101_ after incubation with effector splenocytes from individual mice immunized with (C) Vif_101_ and PADRE (grey, n = 5) or (D) with Vif_101_ alone (black, n = 5). The percentage of specific lysis was calculated as 100×(experimental release-spontaneous release)/(total release-spontaneous release). Background lysis from an unimmunized mouse is shown (open circles). Significant levels are considered >10% lysis at a 50∶1 ratio of effector:target (E:T) cells. One representative experiment out of three. SFU, spot forming units.

We found that when immunizing with the CD8 T cell epitope Vif_101_ together with PADRE Th epitope a high IFN-γ (above 1000 SFU/million cells) towards Vif_101_ was seen in four out of five animals (Figure 2A) whereas when immunizing with Vif_101_ alone a similar response were seen in three out of five animals (Figure 2B).

Using ^51^Cr-release cytolytic assay (Figure 2C+D), we found that in the animals immunized with Vif_101_ together with PADRE two out of five significantly lysed Vif_101_-loaded target cells (Figure 2C), whereas one out of five mice immunized with Vif_101_ alone lysed Vif_101_-loaded target cells (Figure 1D).

Thus, CD8 T cell responses could be induced by the CTL epitope peptides using the CAF01 adjuvants but including a CD4 T helper epitope improved the CTL response.

### Immunization with novel adjuvant CAF01 induces multiple CD8^+^ T cell responses with proliferative capacity

Proliferative capacity is another desirable property of vaccine-induced T cell responses. We therefore evaluated CD8^+^ and CD4^+^ T cell proliferation using CFSE labeling and 5 days *in vitro* stimulation with individual peptides. Here we used multiepitope immunization with a mixture of 7 HIV-1 derived HLA-A2-restricted CTL epitopes (Gag_150_, Gag_433_, Env_67_, Pol_606_, Vpu_66_, Vif_101_ and Vif_23_), plus two HIV-1 specific CD4^+^ T-helper epitopes (Gag_298_ and Env_570_) and one universal CD4^+^ T-helper epitope (PADRE).

**Figure 3:**
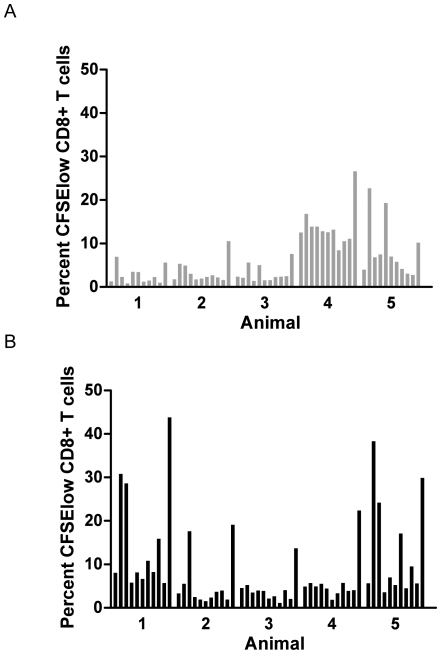
Proliferative CD8^+^ T cell responses induced after immunization with IFA and CAF01. The percentage of proliferating CD3+CD8^+^ splenocytes (having reduced CFSE dye) derived from HLA-A*0201 transgenic HHD mice 10 days after s.c. immunization with a peptide mix consisting of 10 peptides adjuvanted with (A) IFA or (B) CAF01. The background proliferation of non-stimulated (NS) cells, incubated with media alone is shown. The epitope peptides were (from left to right): NS, Gag_150_, Gag_433_, Env_67_, Pol_606_, Vpu_66_, Vif_101_, Vif_23_, Gag_298_ (Th), Env_570_ (Th) and PADRE (Th).

We were able to detect also proliferative CD8^+^ T cells against the CD4^+^ T-helper epitope PADRE in 10 out of 10 immunized mice (Figure 3A+B). In addition up to three minimal CTL epitopes (Gag_150_, Gag_433_, Vif_101_) induced CD8^+^ T cell proliferation in the CAF01 group and up to two epitopes (Gag_150_, Gag_433_) in the IFA group. Moreover PADRE induced CD4^+^ T cell proliferation in 10 out of 10 immunized mice (data not shown).

## Discussion

The new lipophillic adjuvant CAF01 has been found to assist in generation antibody and CD4^+^ T helper responses [5]. Here we show that CAF01 also generate functional CD8^+^ T-lymphocyte responses towards HIV-1 derived minimal CTL epitope peptides in HLA-A*0201 transgenic mice.

Immunological features of induced T cell responses were evaluated using three different assays: IFNγ ELISPOT, ^51^Cr-release cytolytic assay and proliferation using CFSE labeling. The ^51^Cr-release cytolytic assay is recognized as a true functional immunological activity assay where effector splenocytes from the immunized animal are allowed to interact and lyse target cells preloaded with the peptide of interest. Also proliferative capacity is a wanted feature of vaccine induced CD8^+^ T cell-responses. Using all three assays we were able to detect CAF01 induced cellular immune responses to levels comparable with or better than that of incomplete Freund's adjuvant. The level of CD8^+^ T cell responses in the ELISPOT assay can not directly be compared to the level of CD4^+^ T cell responses, as this mouse model contains a very low level of CD8^+^ T cells compared to CD4^+^ T cells [12].

Not many adjuvants are accessible for human use, and those, which can be used in laboratory animal experiments, are often incompatible with clinical trials in humans. CAF01 is a novel adjuvant which has an acceptable safety profile and proved successful in preclinical development of human vaccines against malaria and tuberculosis and are currently in clinical trials [4], [5]. This makes our findings of the induction of functional CTL responses in HLA-A*0201 transgenic mice using CAF01 important.

In addition using multiepitope immunization with CAF01 we were able to induce up to four different specific CD8^+^ T cell proliferative responses in one individual mouse. The most frequently targeted epitopes were the relative dominant CTL epitopes Gag_150_ (T2L), Gag_433_ and the Th epitope PADRE. This dominance pattern was also seen using immunization with dentritic cells as adjuvants pulsed with the same epitopes (data not shown). The fact that the Th epitope PADRE induce CD4^+^ as well as CD8^+^ T cell responses is explained by the fact that this 13-mer, contains also a minimal HLA-A*0201 binding epitope.

We also wanted to evaluate if including a CD4^+^ T cell epitope helped the induction of CTL responses in immunizations using CAF01. Our data show a slight increase of CTL responses when co-immunizing with PADRE. Even though the help from PADRE in this limited set of data might not be convincing, it appears as the trend is that PADRE provides help to CD8^+^ T cells, and at least it does not diminish CTL responses but add CD4^+^ T cell response.

The adjuvantic properties of CAF01 for CD8^+^ T-cell in humans is planned to be tested in a therapeutic HIV-1 phase 1 trial using minimal CD8^+^ CTL and CD4^+^ T cell epitope peptides, and may prove CAF01 as a very attractive adjuvant component in future therapeutic and prophylactic vaccine trials.

## Materials and Methods

### Ethics statement

Animal experiments were approved and done according to the Danish Animal Experimentation Act, based on the Council of Europe Convention ETS 123, on a license granted by the Ministry of Justice.

### HLA-A*0201 transgenic mice

The HHD transgenic mice kindly provided by F.A. Lemonnier, Institut Pasteur, Paris, France, express a transgenic monochain histocompatibility class I molecule in which the C-terminus of the human β_2_-microglobulin (β_2_m) is covalently linked to the N-terminus of a chimeric heavy chain (HLA-A2.1 α1-α2, H-2D^b^ α3-transmembrane and intracytoplasmic domains) [12]. HHD mice are homozygous for the transgene, and H-2D^b−/−^ and β_2_m^−/−^ double knock out, respectively.

### Peptides

Epitope peptides identified as relevant for HIV-1 vaccination [9], [10], [11] were synthesized by Schafer-N, Copenhagen, Denmark. The purity of the peptides was >95%. The CTL epitope petides used in the present study were: Vif_101_(M9L) (GLADQLIHL), Gag_150_(T2L) (RLLNAWVKV), Gag_433_ (FLGKIWPS), Env_67_(V2I) (NIWATHAC), Pol_606_(T9V) (KLGKAGYVV), Vpu_66_(A9V) (ALVEMGHHV), and Vif_23_(I9V) (SLVKHHMYV). As T-helper epitopes we used a Gag_298_ (KRWIILGLNKIVRMY) [14], Env_570_ (VWGIKQLQARVLAVERYLKD) [15], and the universal PADRE (aKXVAAWTLKAAa, X = cyclohexyl alanine, a = D-alanine) [13].

### HLA-A*0201 transgenic mice immunization

Animal experiments were approved and performed in accordance with the legal requirements in Denmark. 6–7 weeks old HLA-A*0201 transgenic mice were injected subcutaneously with 100 µg of the HLA-A2-restricted minimal epitope Vif_101_ peptide plus 120 µg of the synthetic T-helper peptide PADRE or for multiepitope immunizations 20 µg of each of the 10 peptides. Peptides were adjuvanted either by incomplete Freund's adjuvant (IFA) or 250 µg DDA: 50 µg TDB (CAF01). At day 10 after immunization, mice were sacrificed and the splenocytes were recovered and used for CFSE proliferation assay, or re-stimulated *in vitro* with the immunization-peptide, as previously described [9]. After 5 days, the cells were used for cytolytic ^51^Cr-release assay and IFN-γ ELISpot assay.

### IFN-γ enzyme-linked immunospot (ELISPOT) assay

A standard IFN-γ ELISPOT assay was used as previously described [9]. Splenocytes harvested at day 5 of re-stimulation were used in duplicates with a titration of cells ranging from 500,000 to 18,000. Restimulation was done with 10 µg of antigen/mL during 18 hours of incubation at 37°C, 5% CO_2_. ConA (10 µg/ml) was included as positive control. Numbers of specific IFNγ-secreting cells were measured in an ELISPOT reader (Autoimmun Diagnostika GmbH, Strassberg, Germany), analyzed with AiD3.1 S.R software and expressed as numbers of spot-forming units (sfu) per 10^6^ input cells. The cut-off for positivity was 10 sfu (subtracted the negative background of an unimmunized mouse) per 10^6^ splenocytes and at least twice greater than the negative control mean background activity of <5 sfu/10^6^ input cells (spontaneous release) [9].

### Cytotoxic T Lymphocyte assay

A standard cytotoxic ^51^Cr-release assay was used as previously described [9]. Briefly, HHD-EL4S3^-^Rob target cells [12] were mixed with peptide loaded splenocyte effector cells at effector:target cell ratios (E:T) of 100∶1, 50∶1, 25∶1 and 12.5∶1 in either triplicates or 6-replicates. ^51^Cr-release was measured using a microplate scintillation counter (Topcount, NXT, Packard, Boston, USA). Spontaneous and total ^51^Cr-release was measured by adding culture medium or a detergent (Triton X-100) 2% v/v, respectively. The percentage of specific lysis was calculated as 100×(experimental release - spontaneous release)/(total release - spontaneous release). Spontaneous and total release was in the range of 1200 and 9000 counts per minutes, respectively.

### CFSE proliferation assay

For measuring specific proliferation of mouse splenocytes we used a standard CFSE proliferation assay in which splenocytes were labeled with CFSE (5 µM) and stimulated with target peptide (20 µg/ml) and cultured *in vitro* for 6 days before labeling with anti-CD3-APC (BD Biosciences), anti-CD4-PE (BD Biosciences) and anti-CD8-PerCP (BD Biosciences). PHA (10 µg/ml) was included as positive control and media alone as negative control. Data were acquired on a BD LSRII instrument using FACSdiva software (BD Biosciences) and analyzed with FlowJo software (TreeStar). The percentage of proliferating cells (having reduced CFSE dye) was analyzed within CD3^+^CD4^+^ or CD3^+^CD8+ lymphocytes.
